# Cytosinium hydrogen selenite

**DOI:** 10.1107/S1600536814001275

**Published:** 2014-01-22

**Authors:** Radhwane Takouachet, Rim Benali-Cherif, Nourredine Benali-Cherif

**Affiliations:** a Laboratoire des Structures, Propriétés et Interactions InterAtomiques, Université Abbes Laghrour-Khenchela, 40000 Khenchela, Algeria

## Abstract

In the crystal structure of the title salt, C_4_H_6_N_3_O^+^·HSeO_3_^−^, systematic name 6-amino-2-methyl­idene-2,3-di­hydro­pyrim­idin-1-ium hydrogen selenite, the hydrogenselenite anions and the cytosinium cations are linked *via* N—H⋯O, N—H⋯Se, O—H⋯O, O—H··Se and C—H⋯O hydrogen bonds, forming a three-dimensional framework.

## Related literature   

For the crystal structure of cytosine, see: Barker & Marsh (1964 ▶), and of cytosine monohydrate, see: Jeffrey & Kinoshita (1963 ▶). For examples of some inorganic cytosinium salts, see: Mandel (1977 ▶); Bagieu-Beucher (1990 ▶). For examples of the structures of cytosinium salts of organic acids, see: Gdaniec *et al.* (1989 ▶); Smith *et al.* (2005 ▶). For examples of the structure of the hydrogenselenite anion, see: Richie & Harrison (2003 ▶); Wang *et al.* (2006 ▶); Chomnilpan *et al.* (1981 ▶). 
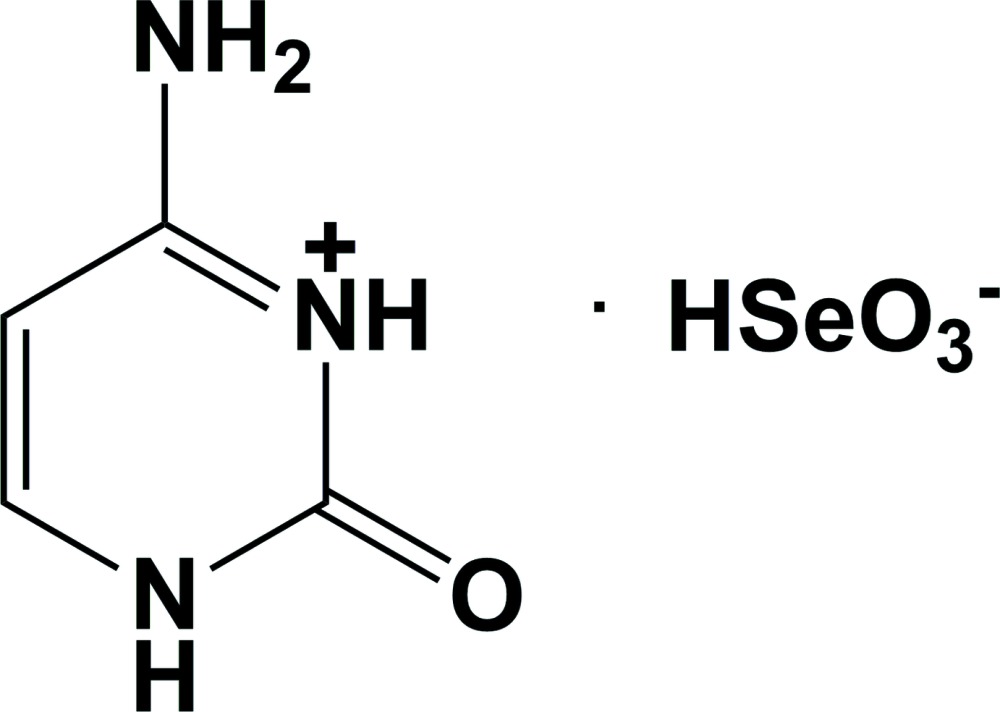


## Experimental   

### 

#### Crystal data  

C_4_H_6_N_3_O^+^·HSeO_3_^−^
*M* *_r_* = 240.09
Orthorhombic, 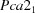

*a* = 7.0051 (3) Å
*b* = 8.6342 (2) Å
*c* = 12.7131 (3) Å
*V* = 768.93 (4) Å^3^
*Z* = 4
Mo *K*α radiation
μ = 4.86 mm^−1^
*T* = 293 K
0.20 × 0.15 × 0.10 mm

#### Data collection  

Nonius KappaCCD diffractometer
Absorption correction: multi-scan (Blessing, 1995 ▶) *T*_min_ = 0.295, *T*_max_ = 0.369
4568 measured reflections
1494 independent reflections
1283 reflections with *I* > 2σ(*I*)
*R*_int_ = 0.067

#### Refinement  

*R*[*F* ^2^ > 2σ(*F* ^2^)] = 0.042
*wR*(*F* ^2^) = 0.098
*S* = 1.04
1494 reflections
125 parameters
7 restraints
H atoms treated by a mixture of independent and constrained refinement
Δρ_max_ = 0.46 e Å^−3^
Δρ_min_ = −0.49 e Å^−3^
Absolute structure: Flack parameter determined using 518 quotients [(*I* ^+^)−(*I* ^−^)]/[(*I* ^+^)+(*I* ^−^)] (Parsons *et al.*, 2013 ▶)
Absolute structure parameter: −0.02 (3)

### 

Data collection: *COLLECT* (Hooft, 1998 ▶); cell refinement: *DENZO* and *SCALEPACK* (Otwinowski & Minor, 1997 ▶); data reduction: *DENZO* and *SCALEPACK*; program(s) used to solve structure: *SIR2004* (Burla *et al.*, 2005 ▶); program(s) used to refine structure: *SHELXL2013* (Sheldrick, 2008 ▶); molecular graphics: *PLATON* (Spek, 2009 ▶); software used to prepare material for publication: *SHELXL2013* and *PLATON*.

## Supplementary Material

Crystal structure: contains datablock(s) Global, I. DOI: 10.1107/S1600536814001275/su2689sup1.cif

Structure factors: contains datablock(s) I. DOI: 10.1107/S1600536814001275/su2689Isup2.hkl

Supporting information file. DOI: 10.1107/S1600536814001275/su2689Isup3.cml

CCDC reference:

Additional supporting information:  crystallographic information; 3D view; checkCIF report

## Figures and Tables

**Table 1:** Hydrogen-bond geometry (Å, °)

*D*—H⋯*A*	*D*—H	H⋯*A*	*D*⋯*A*	*D*—H⋯*A*
N1—H1*A*⋯Se1^i^	0.85 (3)	3.04 (6)	3.789 (9)	148 (9)
N1—H1*A*⋯O3^i^	0.85 (3)	1.93 (3)	2.785 (10)	176 (10)
N2—H2*A*⋯O4^ii^	0.86 (3)	1.97 (5)	2.798 (12)	160 (10)
N3—H3*A*⋯Se1^iii^	0.84 (3)	3.06 (3)	3.896 (12)	174 (7)
N3—H3*A*⋯O2^iii^	0.84 (3)	2.42 (7)	3.126 (12)	141 (8)
N3—H3*A*⋯O4^iii^	0.84 (3)	2.42 (4)	3.196 (17)	152 (8)
N3—H3*B*⋯O3^ii^	0.84 (3)	1.95 (4)	2.772 (12)	166 (12)
O2—H2⋯Se1^iv^	0.81 (3)	2.97 (6)	3.691 (7)	149 (10)
O2—H2⋯O4^iv^	0.81 (3)	1.87 (3)	2.682 (10)	180 (14)
C3—H3⋯O1^v^	0.93	2.46	3.168 (12)	133
C4—H4⋯O2^vi^	0.93	2.31	3.196 (11)	159

Symmetry codes: (i) 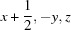; (ii) 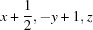; (iii) 
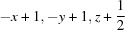
; (iv) 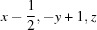; (v) 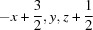; (vi) 
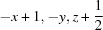
.

**Fig. 1.**
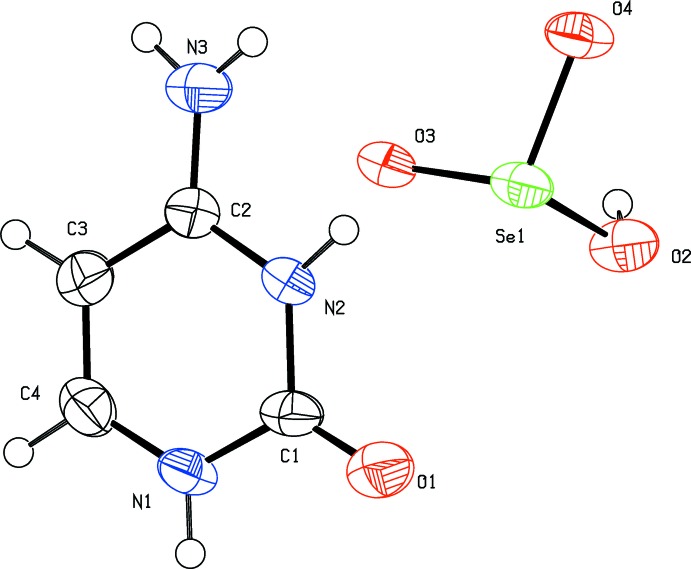
A view of the molecular structure of the title salt, with atom labelling. Displacement ellipsoids are drawn at the 50% probability level.

**Fig. 2.**
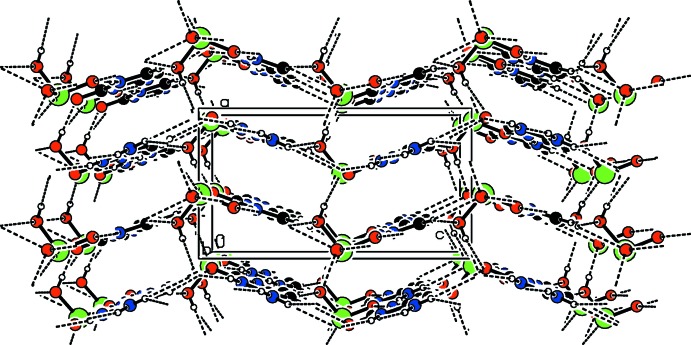
A view along the b axis of the crystal packing of the title compound. The hydrogen-bonds are shown as dashed lines (See Table 1 for details).

## supplementary crystallographic information

### 1. Comment

The crystal structure of cytosine (Barker & Marsh, 1964) and cytosine monohydrate (Jeffrey & Kinoshita, 1963) were determined many years ago. Many inorganic cytosinium salts have been synthesized, including the hydrochloride (Mandel, 1977) and the dihydrogenmonophosphate (Bagieu-Beucher, 1990) salts. Cytosinium salts of organic acids are also common, these include for example, cytosinium trichloroacetate (Gdaniec *et al.*, 1989) and cytosinium 3,5-dinitrosalicylate (Smith *et al.*, 2005). We report herein on the molecular structure of a new cytosinium salt formed by the reaction of cytosine with selenious acid.

The structure of the title salt is illustrated in Fig. 1. The HSeO_3_^-^ ion is pyramidal with two short Se—O bonds, Se1—O3 = 1.634 (8) A° and Se1—O4 = 1.686 (6) A°, and a longer Se—OH bond, Se1—O2 = 1.738 (7) A°. These values are very similar to those described in the literature (Richie & Harrison, 2003; Wang *et al.*, 2006; Chomnilpan *et al.*, 1981). The geometry of this inorganic moiety clearly implies that one proton was transferred from selenious acid to cytosine.

In the crystal, the anions and cations are linked *via* N—H···O/Se, O-H···O/Se and C-H···O hydrogen bonds forming a three-dimensional framework (Table 1 and Fig. 2).

### 2. Experimental

Selenious acid (H_2_SeO_3_) was added to an aqueous solution of cytosine in the stoichiometric ratio 1:1, at room temperature. After four weeks colourless prismatic crystals of the title salt were obtained.

### 3. Refinement

All the H atoms could be located in difference Fourier maps and this was confirmed by plotting difference Fourier maps using the ContourDif routine in PLATON (Spek, 2009). In the final cycles of refinement the NH_2_ distances were restrained to N-H = 0.86 (2) and H···H = 1.33 (2) Å with U_iso_(H) = 1.2U_eq_(N). The OH distance was restrained to O-H = 0.82 (2) Å with U_iso_(H) = 1.5U_eq_(O). The C bound H atoms were included in calculated positions and treated as riding atoms: C-H = 0.93 Å with U_iso_(H) = 1.2U_eq_(C).

### Crystal data

**Table:**

C_4_H_6_N_3_O^+^·HSeO_3_^−^	*D*_x_ = 2.074 Mg m^−^^3^
*M**_r_* = 240.09	Mo *K*α radiation, λ = 0.71073 Å
Orthorhombic, *P**c**a*2_1_	Cell parameters from 5415 reflections
*a* = 7.0051 (3) Å	θ = 3.8–29.5°
*b* = 8.6342 (2) Å	µ = 4.86 mm^−^^1^
*c* = 12.7131 (3) Å	*T* = 293 K
*V* = 768.93 (4) Å^3^	Prism, colourless
*Z* = 4	0.20 × 0.15 × 0.10 mm
*F*(000) = 472	

### Data collection

**Table:**

Nonius KappaCCD diffractometer	1494 independent reflections
Radiation source: fine-focus sealed tube	1283 reflections with *I* > 2σ(*I*)
Graphite monochromator	*R*_int_ = 0.067
ω – θ scans	θ_max_ = 26.0°, θ_min_ = 3.8°
Absorption correction: multi-scan (Blessing, 1995)	*h* = −8→8
*T*_min_ = 0.295, *T*_max_ = 0.369	*k* = −10→10
4568 measured reflections	*l* = −14→15

### Refinement

**Table:**

Refinement on *F*^2^	Hydrogen site location: difference Fourier map
Least-squares matrix: full	H atoms treated by a mixture of independent and constrained refinement
*R*[*F*^2^ > 2σ(*F*^2^)] = 0.042	*w* = 1/[σ^2^(*F*_o_^2^) + (0.0404*P*)^2^ + 0.8215*P*] where *P* = (*F*_o_^2^ + 2*F*_c_^2^)/3
*wR*(*F*^2^) = 0.098	(Δ/σ)_max_ < 0.001
*S* = 1.04	Δρ_max_ = 0.46 e Å^−^^3^
1494 reflections	Δρ_min_ = −0.49 e Å^−^^3^
125 parameters	Extinction correction: *SHELXL*, Fc^*^=kFc[1+0.001xFc^2^λ^3^/sin(2θ)]^-1/4^
7 restraints	Extinction coefficient: 0.018 (4)
Primary atom site location: structure-invariant direct methods	Absolute structure: Flack parameter determined using 518 quotients [(*I*^+^)-(*I*^-^)]/[(*I*^+^)+(*I*^-^)] (Parsons *et al.*, 2013)
Secondary atom site location: difference Fourier map	Absolute structure parameter: −0.02 (3)

### Special details

**Table:**

Geometry. All esds (except the esd in the dihedral angle between two l.s. planes) are estimated using the full covariance matrix. The cell esds are taken into account individually in the estimation of esds in distances, angles and torsion angles; correlations between esds in cell parameters are only used when they are defined by crystal symmetry. An approximate (isotropic) treatment of cell esds is used for estimating esds involving l.s. planes.

### Fractional atomic coordinates and isotropic or equivalent isotropic displacement parameters (Å^2^)

**Table:**

	*x*	*y*	*z*	*U*_iso_*/*U*_eq_	
O1	0.9370 (10)	0.0316 (8)	0.0502 (5)	0.050 (2)	
N1	0.8269 (12)	−0.0384 (8)	0.2127 (7)	0.0392 (19)	
H1A	0.832 (17)	−0.134 (5)	0.194 (8)	0.047*	
N2	0.8534 (12)	0.2200 (8)	0.1669 (6)	0.0363 (17)	
H2A	0.885 (14)	0.300 (8)	0.130 (7)	0.044*	
N3	0.7775 (19)	0.4148 (8)	0.2818 (10)	0.047 (3)	
H3A	0.732 (17)	0.455 (9)	0.337 (5)	0.056*	
H3B	0.817 (16)	0.489 (8)	0.245 (6)	0.056*	
C1	0.8779 (14)	0.0666 (10)	0.1365 (8)	0.037 (2)	
C2	0.7901 (14)	0.2661 (10)	0.2619 (8)	0.036 (2)	
C3	0.7485 (14)	0.1527 (10)	0.3383 (8)	0.041 (2)	
H3	0.7088	0.1799	0.4056	0.049*	
C4	0.7686 (17)	0.0029 (10)	0.3095 (8)	0.041 (2)	
H4	0.7413	−0.0742	0.3583	0.049*	
Se1	0.43127 (11)	0.36795 (7)	0.02452 (11)	0.0378 (4)	
O2	0.2460 (11)	0.3114 (7)	−0.0579 (6)	0.0444 (16)	
H2	0.147 (10)	0.350 (12)	−0.038 (10)	0.067*	
O3	0.3461 (15)	0.3439 (7)	0.1431 (6)	0.054 (2)	
O4	0.4180 (9)	0.5615 (6)	0.0084 (8)	0.0432 (19)	

### Atomic displacement parameters (Å^2^)

**Table:**

	*U*^11^	*U*^22^	*U*^33^	*U*^12^	*U*^13^	*U*^23^
O1	0.063 (4)	0.039 (3)	0.048 (6)	0.004 (3)	0.007 (3)	−0.006 (3)
N1	0.051 (5)	0.022 (3)	0.045 (5)	0.001 (3)	−0.005 (4)	0.003 (3)
N2	0.051 (4)	0.023 (4)	0.035 (4)	0.000 (3)	−0.001 (3)	0.004 (3)
N3	0.062 (8)	0.029 (3)	0.049 (5)	0.002 (5)	0.004 (5)	−0.004 (4)
C1	0.038 (5)	0.024 (4)	0.047 (6)	−0.001 (4)	−0.004 (4)	−0.004 (4)
C2	0.043 (7)	0.030 (4)	0.035 (6)	0.004 (4)	−0.002 (5)	−0.001 (4)
C3	0.050 (6)	0.036 (5)	0.036 (5)	−0.004 (4)	0.000 (4)	−0.002 (4)
C4	0.045 (6)	0.035 (5)	0.042 (6)	−0.003 (4)	−0.003 (5)	0.009 (4)
Se1	0.0425 (5)	0.0233 (4)	0.0475 (5)	0.0031 (3)	−0.0018 (7)	−0.0008 (7)
O2	0.046 (4)	0.035 (3)	0.053 (4)	0.002 (3)	−0.002 (3)	−0.009 (3)
O3	0.098 (6)	0.022 (3)	0.041 (4)	0.005 (3)	−0.001 (4)	0.002 (3)
O4	0.052 (3)	0.023 (3)	0.055 (5)	−0.001 (2)	0.003 (4)	−0.001 (3)

### Geometric parameters (Å, º)

**Table:**

O1—C1	1.211 (11)	N3—H3B	0.84 (3)
N1—C4	1.345 (14)	C2—C3	1.409 (13)
N1—C1	1.374 (13)	C3—C4	1.352 (12)
N1—H1A	0.85 (3)	C3—H3	0.9300
N2—C2	1.346 (11)	C4—H4	0.9300
N2—C1	1.391 (11)	Se1—O3	1.634 (8)
N2—H2A	0.86 (3)	Se1—O4	1.686 (6)
N3—C2	1.312 (12)	Se1—O2	1.738 (7)
N3—H3A	0.84 (3)	O2—H2	0.81 (3)
			
C4—N1—C1	123.3 (7)	N3—C2—C3	122.2 (10)
C4—N1—H1A	121 (7)	N2—C2—C3	118.7 (8)
C1—N1—H1A	115 (7)	C4—C3—C2	117.2 (9)
C2—N2—C1	124.9 (8)	C4—C3—H3	121.4
C2—N2—H2A	110 (7)	C2—C3—H3	121.4
C1—N2—H2A	125 (7)	N1—C4—C3	122.2 (8)
C2—N3—H3A	126 (6)	N1—C4—H4	118.9
C2—N3—H3B	128 (6)	C3—C4—H4	118.9
H3A—N3—H3B	106 (5)	O3—Se1—O4	102.6 (4)
O1—C1—N1	124.3 (9)	O3—Se1—O2	104.3 (5)
O1—C1—N2	122.1 (9)	O4—Se1—O2	99.4 (4)
N1—C1—N2	113.6 (8)	Se1—O2—H2	110 (9)
N3—C2—N2	119.0 (9)		
			
C4—N1—C1—O1	177.3 (10)	C1—N2—C2—C3	1.5 (15)
C4—N1—C1—N2	−3.4 (14)	N3—C2—C3—C4	−179.2 (12)
C2—N2—C1—O1	−179.4 (10)	N2—C2—C3—C4	−2.4 (15)
C2—N2—C1—N1	1.3 (13)	C1—N1—C4—C3	2.7 (17)
C1—N2—C2—N3	178.4 (10)	C2—C3—C4—N1	0.4 (16)

### Hydrogen-bond geometry (Å, º)

**Table:**

*D*—H···*A*	*D*—H	H···*A*	*D*···*A*	*D*—H···*A*
N1—H1*A*···Se1^i^	0.85 (3)	3.04 (6)	3.789 (9)	148 (9)
N1—H1*A*···O3^i^	0.85 (3)	1.93 (3)	2.785 (10)	176 (10)
N2—H2*A*···O4^ii^	0.86 (3)	1.97 (5)	2.798 (12)	160 (10)
N3—H3*A*···Se1^iii^	0.84 (3)	3.06 (3)	3.896 (12)	174 (7)
N3—H3*A*···O2^iii^	0.84 (3)	2.42 (7)	3.126 (12)	141 (8)
N3—H3*A*···O4^iii^	0.84 (3)	2.42 (4)	3.196 (17)	152 (8)
N3—H3*B*···O3^ii^	0.84 (3)	1.95 (4)	2.772 (12)	166 (12)
O2—H2···Se1^iv^	0.81 (3)	2.97 (6)	3.691 (7)	149 (10)
O2—H2···O4^iv^	0.81 (3)	1.87 (3)	2.682 (10)	180 (14)
C3—H3···O1^v^	0.93	2.46	3.168 (12)	133
C4—H4···O2^vi^	0.93	2.31	3.196 (11)	159

Symmetry codes: (i) *x*+1/2, −*y*, *z*; (ii) *x*+1/2, −*y*+1, *z*; (iii) −*x*+1, −*y*+1, *z*+1/2; (iv) *x*−1/2, −*y*+1, *z*; (v) −*x*+3/2, *y*, *z*+1/2; (vi) −*x*+1, −*y*, *z*+1/2.
